# Fungal Metabolomics: A Comprehensive Approach to Understanding Pathogenesis in Humans and Identifying Potential Therapeutics

**DOI:** 10.3390/jof11020093

**Published:** 2025-01-24

**Authors:** Vinicius Alves, Daniel Zamith-Miranda, Susana Frases, Joshua D. Nosanchuk

**Affiliations:** 1 Laboratório de Biofísica de Fungos, Instituto de Biofísica Carlos Chagas Filho, Universidade Federal do Rio de Janeiro, Rio de Janeiro 21941-902, Brazil; viniciusalves@biof.ufrj.br; 2 Department of Microbiology and Immunology, Albert Einstein College of Medicine, Bronx, NY 10461, USA; daniel.zamithmiranda@einsteinmed.edu; 3 Rede Micologia RJ, Fundação de Amparo à Pesquisa do Estado do Rio de Janeiro—FAPERJ, Rio de Janeiro 21040-360, Brazil; 4 Department of Medicine (Infectious Diseases), Albert Einstein College of Medicine, Bronx, NY 10461, USA

**Keywords:** metabolomics, fungal infection, secondary metabolism

## Abstract

Metabolomics has emerged as a transformative tool in the study of microbes, including pathogenic fungi, facilitating the identification of unique metabolic profiles that elucidate their pathogenic mechanisms, host interactions, and treatment resistance. This review highlights key applications of metabolomics in understanding fungal metabolites essential for human virulence, such as mycotoxins produced by various fungal species, including *Aspergillus fumigatus* (gliotoxin, fumagillins) and *Candida* species (phenylethyl alcohol, TCA cycle metabolites), and secondary metabolites that contribute to pathogenicity. It also explores the metabolic adaptations of fungi in relation to drug resistance and biofilm formation, revealing alterations in key metabolic pathways during infection, as seen in *C. albicans* and *C. auris*. Furthermore, metabolomics aids in deciphering host–pathogen interactions, showcasing how fungi like *Cryptococcus neoformans* and *Candida* modify host metabolism to promote survival and evade immune responses. The study of antifungal resistance mechanisms has also benefited from metabolomic approaches, identifying specific metabolite patterns that signify resistance, such as in *Candida albicans* and *Candidozyma (Candida) auris*, and informing new therapeutic strategies. The integration of metabolomics with other omics technologies is paving the way for a comprehensive understanding of fungal biology and pathogenesis. Such multi-omics approaches are crucial for discovering new therapeutic targets and developing innovative antifungal treatments. Thus, the purpose of this review is to provide an overview of how metabolomics is revolutionizing our understanding of fungal pathogenesis, drug resistance, and host interactions, and to highlight its potential for identifying new therapeutic targets and improving antifungal strategies.

## 1. Introduction

The evolution of fungal pathogens remains a central topic of research in major scientific research groups. Although the mechanisms of immune evasion are not fully understood, it is believed that their development began through interactions between fungi and plants, as well as fungi and invertebrates, before interacting with humans [1,2,3]. For example, *Candidozyma (Candida) auris* has emerged as a significant global health threat, with its origins and rapid evolution into a pathogenic form still not fully understood [4,5]. This fungus likely evolved from a common ancestor, diversifying into distinct clades across various geographical regions [5]. Phylogenomic analyses indicate multiple transitions between environmental and human-associated niches, suggesting a more complex evolutionary history rather than a single origin of pathogenicity [6]. *C. auris* can quickly develop adaptive traits, such as multicellular aggregative morphology, through genetic mutations, which enhances its survival in host environments and increases resistance to antimicrobial peptides [7]. The pathogen’s emergence is thought to result from a combination of factors, including climate change, globalization, and a growing population of at-risk individuals [4,6]. Understanding these evolutionary processes is essential for effectively managing *C. auris* infections and anticipating future emerging pathogens.

Beyond their pathogenic potential, fungi have significant biotechnological and industrial applications [8]. A major part of this potential is linked to their ability to produce natural metabolites with unique chemical structures and remarkable versatility [9]. A landmark in medical history was the discovery of the first antibiotic—penicillin, which was derived from *Penicillium notatum* (now classified as *P. chrysogenum*) [10]. Nearly 100 years after the discovery of penicillin, it is well established that fungi possess some of the most advanced secondary metabolite machinery among organisms [11]. These metabolites have been harnessed to develop critical agrochemicals, antibiotics, immunosuppressants, antiparasitic, and anticancer agents, highlighting the substantial impact of fungal-derived compounds [9,12,13,14].

In this context of evolving fungal threats together with the remarkable metabolic plasticity of fungi, understanding the full spectrum of fungal metabolite functions and applications is essential. This knowledge is crucial not only for industrial and ecological contexts but also for exploring new therapeutic applications to address the ongoing challenges of pathogen evolution and immune evasion.

## 2. Metabolomics: Techniques, Applications, and Challenges

**Figure 1:**
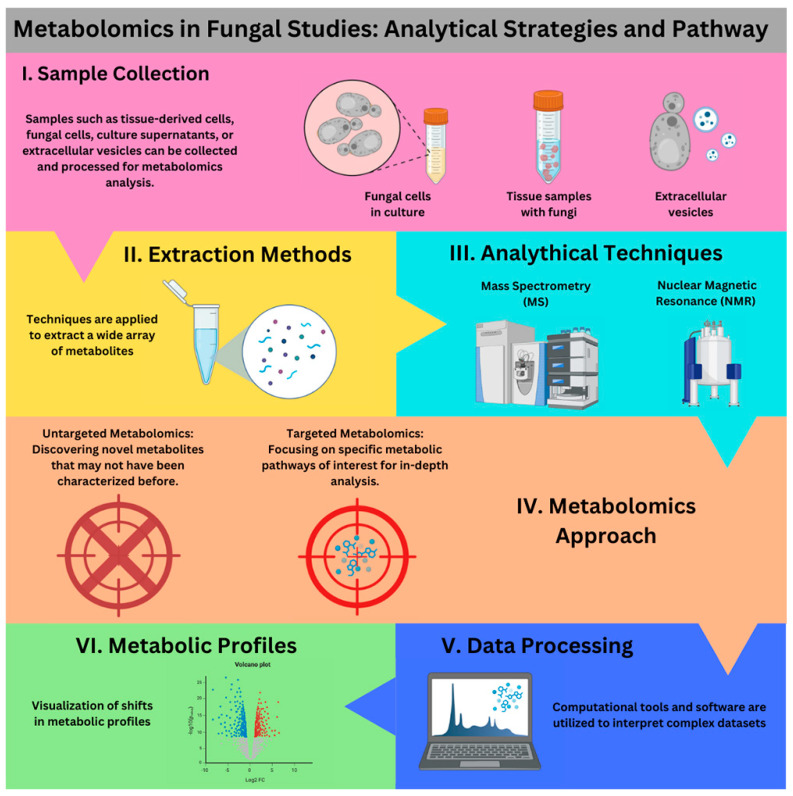
Analytical pathway for metabolomics in fungal research. Samples are collected from in vitro and in vivo environments, followed by extraction methods that isolate primary metabolites (e.g., amino acids) and secondary metabolites (e.g., mycotoxins). Analytical techniques such as mass spectrometry (MS) and nuclear magnetic resonance (NMR) spectroscopy are then employed for identification and structural analysis. The process can incorporate untargeted or targeted metabolomics to explore novel metabolites and specific metabolic pathways. Data processing and statistical analysis involve computational tools to map metabolic pathways and correlate metabolites with biochemical processes. Finally, visualization of metabolic profiles enables tracking of changes during fungal growth or in response to environmental stressors.

Metabolomics is a scientific discipline dedicated to the identification and quantification of chemical compounds within biological samples, as illustrated in Figure 1 [15]. Metabolomics employs a range of advanced analytical techniques, with mass spectrometry (MS) and nuclear magnetic resonance (NMR) being the most widely used platforms [16,17]. In recent years, metabolomics has made significant strides, driven by advances in chromatographic methods, enhanced instrumentation, and the development of sophisticated bioinformatics tools. These innovations have enabled the detection and analysis of hundreds of metabolites in a single experiment [18]. Metabolomics provides crucial insights into cellular pathways, biological mechanisms, and physiological states, making it essential for biomarker discovery, disease diagnostics, and the study of complex biological samples [16,19].

Although this field utilizes various methodologies, mass spectrometry (MS) is frequently favored for its high sensitivity and ability to detect a diverse range of molecules in complex biological samples [20]. Mass spectrometry-based metabolomics is typically categorized into two primary approaches: untargeted and targeted [21]. The untargeted approach aims to identify a wide variety of metabolites, striving to detect as many compounds as possible within a sample [22]. In contrast, targeted metabolomics concentrates on specific classes of molecules, allowing for enhanced precision and sensitivity in the identification and quantification of metabolites of interest [23]. The choice between these approaches largely depends on the research question. When applying metabolomics to a biological sample for the first time, particularly in the absence of prior knowledge, the untargeted approach is often more appropriate. Conversely, when the objective is to detect the presence or production of a specific, well-characterized compound, the targeted approach is more suitable [15,24,25].

Metabolomics offers a comprehensive view of the functionality of biological systems at the time of analysis, often providing a close reflection of the phenotype [26]. Initially, the goal of metabolomics was to identify all small molecules present in biological samples, whether endogenous or exogenous. Nevertheless, limitations in sample processing techniques for mass spectrometry often hinder the complete identification of these molecules [22,27].

Data analysis poses another significant challenge in metabolomics, including issues such as the complexity of metabolite identification, variability in sample matrices, and the limitations of current analytical techniques [27]. A promising strategy to overcome these challenges involves integrating metabolomic data with insights from other omics disciplines, such as proteomics and transcriptomics [28]. However, it is important to note that the use of one technique is not necessarily contingent upon the others.

Metabolomics has emerged as a powerful tool for understanding pathogenic fungi that cause diseases in plants, animals, and humans. By analyzing the unique metabolic profiles of these fungi, researchers gain insights into their pathogenic mechanisms, interactions with hosts, and resistance to treatment.

## 3. Secondary Metabolites and Fungi

Fungi are remarkable organisms known for their capacity to thrive in diverse environments, showcasing remarkable adaptability [29,30]. A striking example of this resilience is the growth of fungi around radioactive areas around and in the Chernobyl powerplant, occurring years after the catastrophic nuclear disaster [31,32]. This survival is largely attributed to the production of melanin, a vital secondary metabolite that provides radiation protection [33]. Melanin, a vital secondary metabolite, plays a critical role in radiation protection for melanized fungi, which exhibit enhanced growth, radiotropism, and improved survival in response to radiation exposure [34,35].

The protective capabilities of melanin against ionizing radiation significantly enhance the survival and growth of fungi in high-radiation environments [36]. These radioprotective properties stem from melanin’s unique chemical composition, its ability to quench free radicals, and its spherical spatial arrangement [37]. Many fungi produce melanin, which contributes to their remarkable resilience against various types of radiation, including UV, gamma, and alpha particles. Notable examples of melanized fungi include *Cryptococcus neoformans*, *Alternaria alternata*, *Cladosporium sphaerospermum*, *Wangiella dermatitidis*, and *Exophiala dermatitidis*, among others [38]. These fungi demonstrate enhanced growth and survival in high-radiation environments [38,39].

Importantly, melanin’s role extends beyond mere radiation shielding; it may also facilitate energy transduction and cellular communication [39]. The adaptive advantages conferred by melanization enable fungi to flourish in extreme conditions, including nuclear reactor sites and space stations [36]. This remarkable resilience is intricately linked to the production of secondary metabolites—compounds synthesized in response to environmental stresses and external stimuli that activate the fungal genome [9].

### 3.1. Biosynthesis Routes

Fungi are remarkable producers of secondary metabolites, with the main metabolic pathways for their production being nonribosomal peptides (NRPs), polyketides, ribosomally synthesized peptides (RiPPs), terpenoids, and alkaloids, as summarized in Table 1 [9]. These compounds, synthesized through complex biosynthetic pathways, exhibit a wide range of biological activities that hold significant potential for various applications.

Nonribosomal peptides (NRPs) are secondary metabolites synthesized by multifunctional enzyme complexes in fungi, independent of ribosomes [40]. These compounds exhibit diverse biological activities, including insecticidal, antibiotic, and antitumor properties [40]. NRPSs contain adenylation, thiolation, and condensation domains, with adenylation domains determining amino acid specificity [41]. Recent advances in fungal NRP research include the discovery of novel compounds and engineering of NRPS assembly lines to generate unnatural products with improved or novel bioactivities [42]. However, challenges remain in efficiently reprogramming these pathways due to their complexity and the large size of NRPSs [42]. Emerging gene disruption, cluster expression, and proteomic strategies are expected to enhance our understanding of fungal NRP biosynthesis [41].

Fungal polyketides are diverse natural products with various biological activities, synthesized by polyketide synthases (PKSs) [43]. These megasynthases produce carbon scaffolds that are further modified by tailoring enzymes, often working iteratively to catalyze multiple reactions [44]. Fungal PKSs are classified into highly reducing (HR-PKSs), non-reducing (NR-PKSs), and hybrid types like PKS-NRPSs and HR-NR PKSs [45]. The biosynthetic programming of fungal PKSs involves complex processes such as starter unit selection, chain length control, and cyclization specificity [46]. Recent research has provided insights into the molecular mechanisms of fungal polyketide biosynthesis, including the formation of reactive poly-β-keto intermediates and their subsequent modifications [46]. Understanding these mechanisms is crucial for discovering new biosynthetic gene clusters and compounds with novel structures [43,47].

Ribosomally synthesized and post-translationally modified peptides (RiPPs) are an emerging class of fungal natural products with diverse bioactivities and unique structural features [48]. The biosynthetic machinery of the first characterized fungal RiPP, ustiloxin B, was identified to contain novel oxidases and side-chain modifications essential for its production [49]. Four distinct families of fungal RiPPs are currently recognized: amatoxins/phallotoxins, borosins, dikaritins, and epichloëcyclins [50]. While their potential benefits to humans are not well explored, the ecological roles of these compounds for the producing fungi are even more poorly understood [50]. Recent genome mining approaches have adapted bacterial tools to identify novel RiPP biosynthetic gene clusters in fungi, including the first report of potential RiPP production in Trichoderma species [51]. The advancements in understanding fungal RiPP biosynthesis and improved genome mining strategies are expected to facilitate more comprehensive exploration of this underexplored class of natural products.

Fungi, particularly Aspergillus and endophytes, are prolific producers of diverse terpenoids and alkaloids with significant bioactive properties [52,53]. These compounds include mono-, sesqui-, di-, sester-, and triterpenoids, as well as terpenoid-alkaloids, which incorporate nitrogen atoms during biosynthesis [52,54]. The biosynthetic pathways typically involve terpene synthases, cyclases, and various tailoring enzymes such as cytochrome P450 monooxygenases and oxidoreductases [55]. Fungal terpenoids and alkaloids exhibit a wide range of biological activities, including antimicrobial properties, making them potential sources for drug development [53]. While Ascomycota have been well studied, less is known about terpenoid biosynthesis in Basidiomycota, despite their production of diverse bioactive compounds [55]. Genome sequencing and bioinformatic methods can facilitate the identification of biosynthetic gene clusters and potentially lead to the discovery of new pharmaceutically relevant fungal terpenoids [55].

### 3.2. Applications of Secondary Metabolites

As shown in Table 2, fungi are prolific producers of bioactive secondary metabolites with diverse applications in pharmaceuticals, agriculture, and cosmetics [56,57]. These compounds include antibiotics, enzyme inhibitors, and immunomodulators [58]. Notable examples are penicillin from *Penicillium notatum* and cephalosporins from *Cephalosporium acremonium*, which target bacterial cell walls [59]. Approximately 40% of FDA-approved new chemical entities are natural products or inspired by them, with many originating from fungi [57]. Advances in genomics and genetics have revealed that fungi possess even greater biosynthetic potential than previously thought, offering promising avenues for drug discovery [60].

Fungal metabolites have emerged as promising sources of antifungal compounds, resulting from evolutionary adaptations to compete with other microorganisms [61]. These secondary metabolites, particularly from endophytic fungi, exhibit diverse chemical structures and potent antifungal properties [62]. They offer potential alternatives to chemical fungicides, addressing concerns about resistance and environmental impact [63]. Fungal metabolites can target various aspects of pathogenic fungi, including biofilm formation, quorum sensing, and cell wall synthesis [64]. Antifungal agents targeting fungal cell walls have gained significant attention due to the increasing prevalence of fungal infections and drug resistance. These agents primarily inhibit the synthesis of cell wall components like glucan, chitin, and mannan [65,66]. Echinocandins, such as caspofungin, inhibit β-1,3-glucan synthesis, while polyoxins and nikkomycins target chitin synthesis [67,68]. Other antifungals like azoles and polyenes inhibit ergosterol biosynthesis. Combinatorial therapies are also being explored to combat resistant fungal strains.

Fungi play a crucial role in sustainable agriculture through their use as biopesticides and growth-promoting agents. Fungal metabolites serve as effective biocontrol agents against pests, diseases, and weeds [69]. Entomopathogenic fungi, such as *Beauveria* and *Metarhizium*, function as mycopesticides, offering host-specific pest control while also promoting plant growth and inducing disease resistance [70]. Various fungal species, including *Penicillium*, *Aspergillus*, and *Trichoderma*, contribute to sustainable agriculture by improving crop growth, soil fertility, and stress management [69]. The development of fungal-based biopesticides offers a promising alternative to chemical pesticides, promoting environmental safety and sustainable agricultural practices.

Fungi offer numerous benefits for skin health and are increasingly utilized in the cosmetics industry. Natural polysaccharides from fungi, such as those from *Tremella fuciformis*, have shown promising effects in wound healing, moisturizing, and anti-aging [71]. *T. fuciformis* extract reduces transepidermal water loss and improves skin hydration [72]. Fungal-derived compounds like kojic acid, β-glucans, triterpenoids, and ceramides are key active ingredients in cosmetic products, offering various benefits such as antioxidant and anti-inflammatory properties [73]. Mushrooms and their extracts have been used in both topical cosmeceuticals and oral nutricosmetics, with ingredients like phenolics, polysaccharides, and vitamins providing anti-aging, skin-whitening, and moisturizing effects [74]. As research progresses, more fungal species are likely to be discovered and utilized in cosmetic applications.

Citrinin and aflatoxins are mycotoxins produced by various *Aspergillus* and *Penicillium* species, posing significant health risks to humans and animals [75]. Citrinin, primarily nephrotoxic and genotoxic, can contaminate a wide range of foods including cereals, fruits, and dairy products [76]. Its toxicity mechanism is not fully understood but may involve oxidative stress or increased mitochondrial membrane permeability [11,77]. Aflatoxins, particularly produced by *A. flavus* and *A. parasiticus*, are carcinogenic and interfere with DNA and RNA synthesis [78]. These mycotoxins can contaminate various agricultural commodities before or after harvest, including corn, peanuts, and cereal grains [75]. Exposure to aflatoxins primarily occurs through ingestion of contaminated food, potentially causing acute or chronic toxic effects such as teratogenicity, mutagenicity, and hepatotoxicity [79]. Proper food storage and effective detection and mitigation strategies are crucial to minimize mycotoxin contamination risks [76,80].

Overall, the diverse roles of fungi in human health, agriculture, and cosmetics, alongside the potential risks associated with certain metabolites, highlight their importance and complexity. This comprehensive understanding of fungal compounds underscores their vast potential and the need for careful consideration in their application.

**Table 2:** Representative therapeutic and bioactive compounds produced by fungi: chemical structures, indications, and mechanisms of action.

Compound	Fungal Producer	Chemical Structure	Indication	Mechanism of Action	Reference
Pharmaceuticals					
Penicillin	*Penicillium chrysogenum*	NRPs	Treatment of bacterial infections	Inhibits bacterial cell wall synthesis	[81]
Cephalosporins	*Cephalosporium acremonium*	NRPs	Various bacterial infections	Inhibit bacterial cell wall synthesis	[59]
Griseofulvin	*Penicillium griseofulvum*	NRPs	Fungal infections of skin, hair, nails	Disrupts fungal cell mitosis	[82]
Caspofungin	*Glarea lozoyensis*	NRPs	Invasive fungal infections	Inhibits beta-(1,3)-D-glucan synthesis	[68]
Micafungin	*Echinos Candida*	NRPs	Treats candidemia and fungal infections	Inhibits beta-(1,3)-D-glucan synthesis	[83]
Voriconazole	Synthetic (derivative of *Fusarium*)	Triazole/Terpenoids	Aspergillosis and candidiasis	Inhibits lanosterol 14-alpha-demethylase	[84]
Lovastatin	*Aspergillus terreus*	Polyketides	Lowers cholesterol levels	Inhibits HMG-CoA reductase	[85]
Ergot Alkaloids	*Claviceps purpurea*	Terpenoids and Alkaloids	Migraines, postpartum hemorrhage	Act on serotonin and dopamine receptors	[86]
Cordycepin	*Cordyceps militaris*	RiPPs	Anticancer and antiviral properties (research)	Inhibits RNA synthesis	[87]
Cyclosporin A	*Tolypocladium inflatum*	NRPs	Immunosuppressant in organ transplantation	Inhibits activation and proliferation of T	[88]
Lentinan	*Lentinula edodes*	RiPPs	Immunomodulator and cancer treatment	Enhances immune function	[89]
Ergotamine	*Claviceps purpurea*	Terpenoids and Alkaloids	Migraine and controls bleeding postpartum	Selective agonist of serotonin receptors (5-HT)	[90]
Mycophenolic acid	*Penicillium brevicompactum*	Polyketides	Immunosuppressant in organ transplant	Inhibits inosine monophosphate dehydrogenase	[91]
Grifolin	*Grifola frondosa*	Terpenoids and Alkaloids	Anticancer and immune-modulating properties	Enhances immune response	[92]
Reishi Mushroom Extract	*Ganoderma lucidum*	Terpenoids and Alkaloids, polysaccharides, peptides	Immune support, anticancer properties	Enhances immune function, antioxidant effects	[93]
Beta-Glucans	*Saccharomyces cerevisiae*	Polysaccharides	Immune modulation, cholesterol-lowering	Enhance immune response and lowers cholesterol absorption	[94]
Tremella Extract	*Tremella fuciformis*	Polysaccharides	Skin hydration, anti-aging	Retains moisture and may promote collagen production	[95]
Pyrrole-2-Carboxylic Acid	*Penicillium* species	Pyrrole derivative	Potential antioxidant and antimicrobial properties	Antioxidant activity and synthesis of essential cellular components	[96]
Triterpenes	*Ganoderma lucidum*, *Lentinula edodes*	Terpenoids and Alkaloids	Anti-inflammatory, anticancer	Modulate various signaling pathways and exhibits multiple biological activities	[97,98]
Antioxidants	Various mushrooms (e.g., *Reishi*, *Chaga*)	Diverse compounds	Protects against oxidative stress	Scavenge free radicals, reduces oxidative damage	[99]
Agriculture					
Trichoderma Metabolites	*Trichoderma* species	Various structures (e.g., proteins, peptides)	Biocontrol agents in agriculture	Antimicrobial activity against phytopathogenic fungi	[100]
Fungal Siderophores	Various fungi (e.g., *Aspergillus*, *Fusarium*)	Small, high-affinity iron-chelating compounds	Iron acquisition for fungi, potential in agriculture	Chelate iron, enhancing nutrient uptake in plants	[101]
Mycopesticides	Various fungi (e.g., *Beauveria*, *Metarhizium*)	Diverse fungal metabolites	Biological pest control	Pest control through infection (e.g., insects)	[102]
Chitosan	Derived from chitin (found in fungal cell walls)	Linear polysaccharide	Antifungal, antibacterial, and wound healing	Enhances plant defense mechanisms, antimicrobial properties	[103]
Beauverin	*Beauveria bassiana*	Peptide	Antifungal activity, potential agricultural use	Disrupts cell function in target organisms	[104]
Fusaric Acid	*Fusarium* species	Polyketides	Plant pathogen control	Inhibits plant growth, disrupts metabolic processes	[105]
Cosmetics					
Essential Oils	*Agaricus bisporus*, *Pleurotus ostreatus*	Terpenoids and Alkaloids	Antimicrobial, antifungal properties	Disrupt microbial membranes, exhibits anti-inflammatory effects	[106]
Tremella Extract	*Tremella fuciformis*	Polysaccharides	Skin hydration, anti-aging	Retains moisture and may promote collagen production	[72]
Chitosan	Derived from chitin in fungal cell walls (e.g., *Zygomycetes*)	Linear polysaccharide	Wound healing, skin care products	Enhances moisture retention and has antimicrobial properties	[107]
Polysaccharides	*Ganoderma lucidum*, *Lentinula edodes*	Various structures (e.g., beta-glucans)	Skin hydration, immune support	Retain moisture, may enhance skin elasticity	[108]
Melanin	*Auricularia auricula*, *Aspergillus* species	Polymer	Skin protection	Scavenges free radicals and protects against UV damage	[109]
Vitamins (e.g., Ergocalciferol)	Mushrooms (e.g., *Agaricus bisporus*)	Secosteroid	Bone health, skin nourishment	Regulate calcium metabolism, may enhance skin health	[110]
Toxics					
Citrinin	*Penicillium* and *Aspergillus* species	Polyketide	Associated with food poisoning	Disrupts cellular processes (exact mechanism not well defined)	[111]
*Fusarium* Toxins (e.g., fumonisins, zearalenone, and trichothecenes)	*Fusarium species* (e.g., *Fusarium graminearum*, *Fusarium verticillioides*, *Fusarium culmorum)*	Fumonisins: long-chain amines; Zearalenone: non-steroidal compound; Trichotecenes: sesquiterpenoid	Carcinogenic effects (fumonisins), reproductive toxicity (zearalenone), mycotoxicosis and immune suppression (trichotecenes)	Fumonisins: inhibit ceramide synthase; Zearalenone: acts as pseudo-estrogen, interfering with hormonal signaling; Trichotecenes: inhibit protein synthesis.	[112]
Paxillus involutus Toxin	*Paxillus involutus*	Complex polysaccharides	Associated with food poisoning	Causes hemolysis and damages kidney tissue	[113]
Aflatoxins	*Aspergillus flavus*, *Aspergillus parasiticus*	Family of related compounds	Associated with food poisoning	Interfere with DNA and RNA synthesis	[78]

## 4. Applications of Metabolomics in Research on Pathogenic Fungi

### 4.1. Identification of Fungal Metabolites

As detailed above, mass spectrometry (MS) is a primary technique for the identification of diverse metabolites produced by pathogenic fungi [27], but it has limitations when analyzing complex mixtures. To overcome these challenges, MS is often combined with chromatographic methods like high-performance liquid chromatography (HPLC), gas chromatography (GC) or UV spectroscopy (UV) [114]. Given the current gaps in knowledge regarding fungal metabolites, particularly in pathogenic fungi, the untargeted metabolomics approach is particularly advantageous, as it facilitates the discovery of unknown metabolites that may serve as new targets for research [22].

Studies have generated significant insights into the secondary metabolites produced by *A. fumigatus* and their roles in pathogenicity. Researchers have identified numerous bioactive compounds, including gliotoxin, fumagillins, and pseurotins, which are involved in virulence and immune suppression [115,116]. These metabolites help *A. fumigatus* adapt to various microenvironments in the host, such as hypoxic conditions in the lung [117]. Some compounds, like hydroxy-(sulfooxy) benzoic acid, may possess anti-inflammatory properties [118]. Additionally, a novel tetrapeptide named aspergitide was discovered, which is specific to *Aspergillus* species and could potentially be used for laboratory diagnosis of aspergillosis [118]. The diverse array of secondary metabolites produced by *A. fumigatus* not only aids in its environmental survival but also contributes to its success as an opportunistic pathogen, highlighting the importance of these compounds in both ecological and clinical contexts [115].

*Fusarium* species are known plant pathogens that produce mycotoxins, including fusaric acid, which has phytotoxic properties [119,120]. Notably, environmental stress can increase fusaric acid production. Metabolomic approaches have been used to identify and characterize bioactive metabolites produced by *Fusarium*, with quinones and non-ribosomal peptides being among the most frequently characterized compounds [121]. Biological control using antagonistic microorganisms, such as *Trichoderma* sp., has also been explored as an alternative to agrochemicals for managing *Fusarium*-induced diseases, particularly in tomato plants [122,123]. Additionally, these biocontrol agents possess plant growth-promoting properties, including phosphate solubilization and production of siderophores, indole acetic acid, and proteases [122]. Understanding the factors influencing mycotoxin production and exploring natural control methods can help reduce toxin presence and improve food utilization.

Recent studies have explored the metabolic profiles of various *Candida* species, including *C. albicans*, *C. auris*, and *C. glabrata*, using gas chromatography-mass spectrometry techniques. These analyses have identified numerous metabolites involved in virulence, biofilm formation, and drug resistance. For instance, *C. auris* secretes hyphae-inhibiting metabolites like phenylethyl alcohol, which is also abundant in other *Candida* species [124,125]. Metabolomic profiling of *C. albicans* biofilms revealed differential production of metabolites involved in the TCA cycle, lipid synthesis, and amino acid metabolism compared to planktonic cells [126]. Furthermore, metabolomic studies on azole-resistant *C. albicans* strains identified alterations in amino acid metabolism, the TCA cycle, and phospholipid composition, suggesting a metabolic shift contributing to drug resistance [127]. These findings provide insights into the metabolic processes underlying *Candida* virulence and drug resistance, potentially guiding the development of new antifungal strategies.

*Cryptococcus neoformans*, the leading cause of fungal meningitis, produces mannitol, which is vital for its virulence and stress tolerance. The synthesis and accumulation of mannitol enables the fungus to endure heat and osmotic stress, thereby enhancing its pathogenicity in mouse models [128]. Additionally, the inositol catabolic pathway plays a significant role in *C. neoformans* virulence by influencing capsule growth and structure [129]. Transcriptional analyses of *C. neoformans* internalized by amoebae and macrophages have identified genes that may contribute to host adaptation, including PTP1, a polyol transporter protein [130]. These insights suggest that the virulence of *C. neoformans* has evolved through interactions with environmental predators, such as protozoa. A comprehensive understanding of the molecular and biochemical mechanisms underlying *C. neoformans* virulence factors—such as capsule formation, melanin production, and polyol metabolism—is essential for developing innovative strategies for disease control [131]. This emphasizes the importance of metabolomics in identifying and characterizing these critical metabolites, paving the way for new therapeutic approaches.

Fungal metabolites play a significant role in carcinogenesis by influencing various biological pathways that drive tumor initiation, progression, and metastasis. Key fungal toxins, such as aflatoxins and ochratoxin A, have been implicated in cancer development through bioactivation processes that lead to the formation of carcinogenic metabolites capable of interacting with DNA. Aflatoxin B1 (AFB1), for example, is metabolized to form an epoxide that can cause DNA damage, triggering neoplastic transformation [132]. Similarly, ochratoxin A (OTA) undergoes bioactivation to form DNA adducts, which may also contribute to its carcinogenic potential [133]. These metabolites can induce genetic, epigenetic, and metabolic changes that disrupt normal cellular functions, promoting the initiation and progression of cancer. Moreover, fungal dysbiosis, often linked to microbial infections, can alter the tumor microenvironment and immune responses, affecting tumor development and treatment outcomes, particularly in immunocompromised individuals [134]. The relationship between fungal toxins and cancer is evident in several cancers, including those of the liver, esophagus, colon, and lung, with fungal infections responsible for initiating 2.2 million new cancer cases globally [135]. Despite the promising anticancer effects of some fungal metabolites in preclinical studies, such as their potential to modulate the NF-kappaB pathway and influence inflammation and metastasis [136], these compounds have not yet reached clinical application. Understanding the complex interplay between fungal toxins, their bioactivation, and their impact on carcinogenesis is crucial for developing novel cancer prevention and therapeutic strategies.

Additionally, metabolomics can track specific metabolite patterns that vary during different infection stages or in response to environmental stressors [26]. These patterns offer insights into how fungi adjust their virulence and survival mechanisms, uncovering new aspects of their biology.

### 4.2. Host–Pathogen Interaction Mechanims

Metabolomics provides profound insights into the interactions between pathogenic fungi and their hosts by comparing metabolic profiles from both organisms. Upon invasion, fungi can significantly alter the host’s metabolism, producing substances that inhibit the host’s defenses or modifying cellular metabolism to promote fungal growth [137].

Recent studies have revealed intricate metabolic interactions between *C. albicans* and the host immune system. *C. albicans* can significantly alter the host’s metabolic profile, impacting the immune response. For example, the fungus can downregulate IL-17 production in human blood mononuclear cells by modulating tryptophan metabolism [138]. Furthermore, *C. albicans* induces the activity of arginase-1 in macrophages, which reduces nitric oxide production and impairs the host’s ability to eradicate the fungus [139]. Additionally, stress during infection can amplify metabolic changes, resulting in greater tissue damage and promoting enhanced fungal colonization [140]. Both host immune cells and *C. albicans* adapt their metabolic pathways in response to infection; for instance, macrophages upregulate glucose uptake and aerobic glycolysis. While the fungus modifies its metabolism to thrive in hostile environments, such as within phagolysosomes, it shifts to glycolysis, rapidly depleting glucose from the medium and ultimately inducing macrophage cell death [141].

*Talaromyces* (formerly *Penicillium*) *marneffei* is an opportunistic pathogenic fungus causing severe infections in immunocompromised individuals, particularly HIV-infected patients in Southeast Asia [142]. The infection is considered an AIDS-defining illness in endemic regions, with severity correlating to the patient’s immune status [143]. The fungus adapts to intracellular survival by altering its metabolism, upregulating genes involved in the glyoxylate cycle while downregulating glycolytic genes when internalized by macrophages [144]. This metabolic shift allows T. marneffei to persist within host cells by utilizing alternative carbon sources.

Recent studies have explored the interactions between *Mucor circinelloides* and the immune system. *M. circinelloides* can evade macrophage-mediated killing by blocking phagosomal maturation and escaping from phagocytes [145]. The fungus produces various metabolites, including unsaturated fatty acids, which may influence its pathogenicity [146]. Metal ions play a crucial role in *M. circinelloides* growth and lipid accumulation, with Mg and Zn being essential for metabolic activity [147]. In vivo studies using zebrafish larvae have revealed tissue-specific innate immune responses to *M. circinelloides* infection. Macrophages and neutrophils are rapidly recruited to infection sites, forming phagocyte clusters that may contribute to spore dissemination. Interestingly, *M. circinelloides* spores fail to activate pro-inflammatory gene expression in the early stages of infection, with only a weak response observed after 24 h in certain infection models [148].

Understanding these metabolic interactions holds promise for the development of novel antifungal therapies aimed at disrupting these pathways and restoring effective immune responses.

### 4.3. Treatment Resistance

The rise of antifungal resistance poses significant challenges in both medicine and agriculture [149,150]. Understanding shifts in the metabolome are crucial for elucidating resistance mechanisms and identifying associated biomarkers [151]. By examining the metabolic profiles of pathogenic fungi exposed to various antifungals, researchers can detect metabolite alterations indicative of resistance.

As noted above, *C. auris* is an emerging multidrug-resistant pathogenic fungus causing severe invasive infections with high mortality rates [152]. It demonstrates resistance to multiple antifungals, particularly fluconazole and amphotericin B [153]. Resistance mechanisms include mutations in ERG genes, overexpression of efflux pumps, and alterations in FKS1, FCY2, FCY1, and FUR1 genes [153]. Metabolomic studies have identified changes in metabolic profiles associated with resistance, such as the secretion of hyphae-inhibiting metabolites like phenylethyl, benzyl, and isoamyl alcohols [124]. *C. auris* also produces biofilm-forming metabolites like tyrosol, which may contribute to its virulence [124]. The fungus poses a significant nosocomial threat, requiring improved infection control measures in healthcare settings [154]. Accurate identification remains challenging, potentially leading to underestimation of its prevalence [155].

The development of azole resistance in *C. albicans* involves complex metabolic reprogramming. Metabolomic profiling revealed significant alterations in amino acid metabolism, the tricarboxylic acid cycle, and phospholipid metabolism during resistance development [127]. Proteomic analysis identified changes in energy metabolism-related proteins, suggesting a metabolic shift contributes to fluconazole resistance [156]. Lipidomic studies of azole-resistant isolates showed unexpected diversity in lipid profiles, with fluctuations in phosphatidylserine, mannosylinositolphosphorylceramides, and sterol esters indicating their role in maintaining lipid homeostasis [157]. Furthermore, gradual changes in plasma membrane microdomain-specific lipids and mitochondrial phosphatidylglycerol were observed as resistance developed [158]. These studies collectively demonstrated the multifactorial nature of azole resistance in *C. albicans*, involving metabolic shifts, lipid profile alterations, and potential crosstalk between mitochondrial function, cell wall integrity, and drug resistance mechanisms.

Studies have explored its metabolic adaptations during infection and treatment. *H. capsulatum* forms biofilms, which show reduced susceptibility to itraconazole and amphotericin B compared to farnesol [159]. Proteomic analyses reveal differential metabolic shifts between mycelial and yeast forms, with yeast cells showing higher abundance of tricarboxylic acid cycle and stress response proteins [160]. Mebendazole, a potential alternative treatment, inhibits *H. capsulatum* growth by disrupting mitochondrial function and cytoskeleton structure [161]. The fungus adapts to various environmental conditions during infection, modulating gene expression and phenotypic traits to survive in harsh environments, including within macrophages [162]. These studies provide insights into *H. capsulatum*’s metabolic adaptations and potential new treatment strategies for histoplasmosis.

### 4.4. Discovery of Therapeutic Targets

Metabolomics have been instrumental in discovering new therapeutic targets by mapping active metabolic pathways in pathogenic fungi [24,163,164]. If a specific metabolic pathway is crucial for toxin production or host invasion, inhibiting that pathway could significantly reduce fungal virulence. Metabolomics facilitates the detailed analysis of metabolic changes in response to various conditions and treatments, aiding in the identification of such targets [165,166].

For example, fungi like *C. albicans* can manipulate the host’s iron metabolism to secure essential nutrients, evident through changes in iron profiles or related metabolites [167,168,169]. *C. albicans* possesses a sophisticated iron acquisition system crucial for its commensal-pathogenic lifestyle [169]. During systemic infection, *C. albicans* activates the high-affinity iron permease Ftr1 to acquire iron from the host, while modulating iron uptake in the gut to prevent toxicity [168,170]. The fungus contains multiple permeases and ferroxidases, forming a dynamic iron transport system with flexible partnerships and distinct expression patterns [169]. This system allows *C. albicans* to adapt to varying iron availability in different host niches [171]. The transcription circuit controlling iron uptake involves Sef1, Sfu1, and Hap43, which regulate gene expression for iron acquisition in the bloodstream and iron toxicity prevention in the gut [170]. This complex iron homeostasis mechanism is essential for *C. albicans* virulence and commensalism, highlighting potential targets for medical intervention in infections [168,169].

*H. capsulatum* adapts to host environments through various metabolic strategies. Studies have revealed the fungus’s ability to modulate its lipid metabolism, with potential targets for antifungal drug development identified in fatty acid desaturation and sphingolipid biosynthesis pathways [172]. *H. capsulatum*’s response to iron limitation, a key host defense mechanism, involves alterations in glycolysis, TCA cycle, and protein synthesis [173]. The fungus acquires iron from host sources using hydroxamate siderophores and ferric reductases [174]. As an intracellular pathogen, *H. capsulatum* survives within macrophages by regulating phagosomal pH [174]. The fungus’s ability to adapt to various environmental conditions, including those encountered during infection, is crucial for its pathogenicity [162]. These metabolic adaptations offer potential targets for developing new therapeutic approaches against *H. capsulatum*.

*C. neoformans* adapts its metabolism during host infection to enhance virulence. Upon initial lung infection, *C. neoformans* upregulates genes for central carbon metabolism, acetyl-CoA production, and alternative carbon source utilization [175]. The fungus employs both peroxisomal and mitochondrial β-oxidation pathways to utilize host lipids, influencing growth and virulence [176]. A Cryptococcus genome-scale metabolic model revealed that steroid and amino acid metabolisms are potential drug targets [177]. Key virulence factors, including capsule production, melanin synthesis, and stress responses, are regulated by specific transcription factors and integrated with metabolic adaptation [178]. The ACS1 gene, encoding acetyl-CoA synthetase, and the SNF1 gene, involved in alternative carbon source utilization, both contribute to *C. neoformans* virulence [175]. Notably, pathways involved in lipid metabolism and amino acid synthesis emerged as promising candidates, offering new avenues for antifungal therapies aimed at mitigating the impact of this devastating pathogen.

*Coccidioides immitis* and *C. posadasii* are dimorphic fungal pathogens causing coccidioidomycosis in arid regions of the Americas [179]. Comparative genomics and transcriptomics have revealed insights into their biology and virulence. Gene expression differences between saprobic and parasitic phases highlight the importance of cell surface-associated genes, particularly those related to chitin, and virulence factors like alpha (1,3) glucan synthase and SOWgp [180]. Evolutionary changes in Coccidioides metabolism genes, membrane-related proteins, and potentially antigenic compounds suggest adaptation to animal hosts rather than soil saprophytism [181]. Antifungal resistance mechanisms include target mutation, efflux systems, and pleiotropic drug responses. Novel drug targets being investigated include metabolic pathways such as trehalose and amino acid metabolism. Development of new antifungal agents, including nanostructured formulations and repositioned drugs, as well as vaccines, may improve treatment outcomes for fungal infections [182].

## 5. Challenges and Future Perspectives

### 5.1. Metabolomics and Other Omics Technologies in Fungal Research

Despite its potential, metabolomics faces significant challenges, including the complexity of biological samples and the difficulty in identifying small metabolites [183,184]. While mass spectrometry is a powerful tool, it may struggle to detect certain metabolites due to interferences and resolution limits [17].

Integrating multi-omics approaches, including transcriptomics, proteomics, lipidomics, and metabolomics, has significantly enhanced our understanding of fungal biology and pathogenesis [185,186]. These techniques provide comprehensive insights into fungal metabolic processes, virulence mechanisms, and responses to environmental changes [187].

The integration of genomics and metabolomics has provided important insights into the pathogenicity of *A. fumigatus*. Genome sequencing has revealed biosynthetic gene clusters associated with secondary metabolites, such as gliotoxin and fumitremorgin, which are considered potential virulence factors of fungi in human infections, particularly in immunocompromised individuals. [188,189]. When challenged by human dendritic cells, the fungus activates alternative metabolic pathways for amino acids and carbon, as well as stress-regulating enzymes [190]. Comparative genomics also suggests that closely related species, such as *A. novofumigatus*, may possess similar pathogenic potential [189].

Recent studies have also integrated lipidomics and metabolomics to investigate the role of lipids in the pathogenicity of *C. neoformans*. Lipids act as signaling molecules and regulate pathogenic traits in the fungus [191]. Spectroscopic analyses of lipid extracts revealed complex sphingolipid, sterol, and phospholipid profiles, providing insights into their biosynthesis and metabolism [192]. Ceramide synthase has been identified as a potential target for antifungal therapy, playing a crucial role in *C. neoformans* virulence [193]. The fungus adapts to various host environments by remodeling its central carbon metabolism, expressing specific nutrient acquisition systems, and responding to hypoxia. These adaptive mechanisms are regulated by transcription factors that also control the expression of major virulence factors, highlighting the integrated nature of metabolism and virulence in *C. neoformans* [178].

The integration of transcriptomics and metabolomics has been key to understanding *C. albicans* adaptation during infection. Metabolic adaptation significantly impacts *C. albicans* pathogenicity, affecting stress resistance, antifungal susceptibility, and the expression of virulence factors [194]. Techniques like RNA-Seq and LC-MS have mapped transcriptional and metabolic reprogramming in heterogeneous *C. albicans* isolates, revealing complex regulation of gene expression and metabolic pathways in response to environmental stimuli [195]. Microarray technology has been used to identify infection-associated genes and capture genome-wide transcriptomic portraits under different conditions [196], uncovering pathways and genes involved in biofilm formation and drug resistance, critical virulence traits of *C. albicans* [197].

The combination of proteomics and metabolomics has enhanced our understanding of mycotoxin biosynthesis regulation in pathogenic fungi like Fusarium species. Metabolomics is essential for deciphering the regulatory mechanisms of mycotoxin production, exploring the chemical interactions between toxigenic fungi and their environment, and analyzing multiple mycotoxins simultaneously [198]. These omics approaches have expanded knowledge of the molecular processes regulating mycotoxin production and fungal adaptation to environmental stresses [199]. Research has revealed intricate transcriptional and epigenetic regulation of mycotoxin biosynthesis gene clusters, linking environmental factors such as nitrogen, carbon, and pH to mycotoxin production regulation [200]. These integrated approaches help identify regulatory networks controlling mycotoxin production, which can inform strategies to mitigate fungal contamination in crops.

Metabolomics, the newest approach, is gaining popularity for its potential to dissect various aspects of pathogens and diseases [186]. Multi-omics strategies have also been crucial in elucidating the biology of fungal extracellular vesicles, revealing their diverse functions from cell wall biosynthesis to virulence factors [201]. These advanced techniques are now being applied to study antifungal resistance, identify disease biomarkers, and develop potential vaccines, paving the way for improved diagnostics and therapies for fungal infections [201].

### 5.2. Advancements in Metabolomics Software and Untargeted Workflows

Recent advancements in metabolomics software have significantly enhanced untargeted workflows, enabling more accurate data processing, annotation, and visualization in MS- and NMR-based analyses [202]. Tools like UmetaFlow, a computational workflow for untargeted metabolomics, combine algorithms for data pre-processing, spectral matching, and molecular predictions, improving feature detection and annotation accuracy [203]. Additionally, software packages like MZmine 2 perform well in relative quantification and marker selection [204], while integrating 2D NMR metabolomics databases has improved metabolite identification [205]. These innovations are crucial for addressing the complexities of untargeted metabolomics and enabling researchers to manage increasingly large and intricate datasets.

Recent years have also seen an explosion in new metabolomics software tools, with over 85 new resources, packages, and databases introduced in 2020 alone [206]. These tools range from data pre-processing and statistical analysis to workflow management, catering to various platforms, including LC-MS, GC-MS, and NMR [207]. Notably, the XCMS software family has expanded to incorporate specialized features for isotope labeling and multi-group comparisons [208]. These developments are essential for keeping pace with technological advances in mass spectrometry and spectroscopy, supporting applications in precision medicine, drug discovery, and environmental studies [206]. Continued updates and improvements in these tools are critical to addressing the evolving demands of metabolomics research.

Neural networks, a class of machine learning algorithms, are increasingly utilized in metabolomics to enhance data analysis, improve metabolite identification, and facilitate the discovery of biomarkers [209,210]. These networks excel in handling complex, high-dimensional data, offering advantages over traditional methods by predicting compound fingerprints from MS/MS spectra and classifying metabolic profiles with greater accuracy [211]. In fungal research, neural networks, particularly deep neural networks (DNNs) and frameworks like MiMeNet, have shown promise in predicting metabolite abundances, uncovering microbe–metabolite relationships, and identifying key biomarkers [212,213]. The integration of neural networks with techniques like NMR-based metabolomics further improves metabolite classification and quantification, providing more robust tools for fungal metabolite discovery [214]. As these technologies continue to evolve, neural networks hold great potential to accelerate the identification of novel bioactive compounds, facilitate the exploration of complex metabolic pathways, and ultimately drive drug discovery and biotechnological innovations in fungal research [215,216,217,218].

Together, these advancements are not only refining the analysis of complex metabolomics datasets but also opening new possibilities for large-scale screenings, especially in fungal research, where understanding metabolic networks and pathogen–host interactions is increasingly important.

### 5.3. Addressing the Fundamental Challenges in Fungal Metabolomics for Pathogenic Fungi

Despite the significant advancements in fungal metabolomics, several fundamental challenges remain, particularly in the study of pathogenic fungi. One of the primary obstacles is the complexity and variability of fungal metabolomes. The metabolic profiles of fungi can differ widely depending on the species, strain, environmental conditions, and even the host they infect [219,220]. This variability makes it difficult to establish standardized protocols for metabolite identification and quantification across different fungal species, hindering the reproducibility and comparability of results. Furthermore, the chemical diversity of fungal secondary metabolites—many of which are implicated in virulence, host interactions, and drug resistance—poses a significant challenge for accurate detection and characterization [221]. Many of these metabolites are produced in low quantities or are chemically similar to other compounds, making them difficult to identify using current technologies [222].

Another major challenge is the lack of comprehensive and curated databases for fungal metabolites, particularly those associated with pathogenicity. Unlike well-established metabolomic databases for model organisms, the resources available for fungi, especially pathogenic species, are still in their infancy [218,223]. This limits our ability to fully understand the roles of specific metabolites in virulence, drug resistance, and biofilm formation. Moreover, the translation of metabolomics data into clinically relevant outcomes, such as the identification of novel therapeutic targets or biomarkers for disease diagnosis, remains a complex task [224,225].

To address these challenges, future research must focus on developing more refined analytical techniques that can accurately capture the full diversity of fungal metabolites, particularly those involved in pathogenicity. Advances in high-resolution mass spectrometry, as well as the integration of multi-omics approaches, hold great promise for overcoming these barriers [226]. Additionally, the creation of more extensive, publicly accessible metabolite databases specific to pathogenic fungi will be crucial for enhancing the reproducibility and applicability of metabolomic studies [227]. With these advancements, we can begin to bridge the gap between basic fungal metabolomics research and its clinical applications, ultimately leading to the development of more effective antifungal therapies and diagnostic tools.

## 6. Conclusions

In summary, metabolomics is an invaluable tool for studying pathogenic fungi, offering comprehensive insights into their metabolic profiles, which are critical for understanding mechanisms of pathogenesis, host interactions, and resistance to treatment. By analyzing the complex networks of metabolites produced by these fungi, we gain a clearer understanding of how they adapt to host environments, evade immune defenses, and resist antifungal therapies. With continuous advancements in metabolomic technologies, such as improved high-throughput techniques, sophisticated bioinformatics tools, and enhanced analytical platforms, the potential for discovering novel biomarkers, identifying new therapeutic targets, and developing more effective antifungal treatments is immense.

As fungal infections become increasingly prevalent, particularly among immunocompromised individuals, the application of metabolomics promises to transform our approach to diagnosis and treatment, enabling more personalized and effective clinical strategies. The integration of metabolomics with other ’omics’ disciplines—such as genomics and proteomics—further enriches our understanding of fungal biology and pathogenesis. Looking ahead, the synergy between multi-omics approaches will be crucial for pioneering innovative, multi-dimensional strategies to combat these infections. Future directions hold great promise for large-scale screenings of fungal pathogens, providing deeper insights into the metabolomic landscapes that drive pathogenicity and resistance, ultimately contributing to more precise and targeted therapeutic interventions.

## Figures and Tables

**Table 1:** Classification, characteristics, and biosynthesis of major fungal secondary metabolites.

Compound Class	Description	Characteristics
Nonribosomal Peptides (NRPs)	Secondary metabolites produced by multifunctional enzyme complexes in fungi, independent of ribosomes.	Bioactive compounds with insecticidal, antibiotic, and antitumor properties. Biosynthesis catalyzed by NRPSs with adenylation, thiolation, and condensation domains.
Polyketides	Diverse natural compounds produced by polyketide synthases (PKSs), with a broad range of biological activities.	PKSs are megaenzymes that form carbon scaffolds, modified by tailoring enzymes. Types: HR-PKSs, NR-PKSs, PKS-NRPSs, HR-NR PKSs.
Ribosomally Synthesized and Post-Translationally Modified Peptides (RiPPs)	Emerging fungal compounds with diverse bioactivities and unique structural features, synthesized ribosomally and modified post-translationally.	Includes amatoxins, borosins, dikaritins, and epichloëciclines. Their ecological roles are poorly understood, but they hold significant biological potential.
Terpenoids	Terpene-derived compounds produced by fungi with various significant biological activities.	Includes mono-, sesqui-, di-, sester-, and triterpenes, as well as terpenoid-alkaloids. Biosynthesis involves terpene synthases, cyclases, and enzymes like P450 monooxygenases.
Alkaloids	Nitrogen-containing compounds produced by fungi, with various bioactive properties.	Many fungal alkaloids exhibit antimicrobial and therapeutic activity. Biosynthesis typically involves cyclization and post-biosynthesis modifications by a range of enzymes.

## Data Availability

The data in this study are available in the presented manuscript.
